# Hand Focused Upper Extremity Rehabilitation in the Subacute Phase Post-stroke Using Interactive Virtual Environments

**DOI:** 10.3389/fneur.2020.573642

**Published:** 2020-11-26

**Authors:** Alma S. Merians, Gerard G. Fluet, Qinyin Qiu, Mathew Yarossi, Jigna Patel, Ashley J. Mont, Soha Saleh, Karen J. Nolan, AM Barrett, Eugene Tunik, Sergei V. Adamovich

**Affiliations:** ^1^Department of Rehabilitation and Movement Sciences, School of Health Professions, Rutgers Biomedical and Health Sciences, Newark, NJ, United States; ^2^Movement Neuroscience Laboratory, Department of Physical Therapy, Movement and Rehabilitation Science, Bouve College of Health Sciences, Northeastern University, Boston, MA, United States; ^3^SPIRAL Group, Department of Electrical and Computer Engineering, Northeastern University, Boston, MA, United States; ^4^Department of Biomedical Engineering, New Jersey Institute of Technology, Newark, NJ, United States; ^5^Center for Mobility and Rehabilitation Engineering Research, Kessler Foundation, West Orange, NJ, United States; ^6^Department of Physical Medicine and Rehabilitation, Rutgers New Jersey Medical School, Newark, NJ, United States; ^7^Center for Stroke Rehabilitation Research, Kessler Foundation, West Orange, NJ, United States; ^8^Department of Bioengineering, College of Engineering, Northeastern University, Boston, MA, United States; ^9^Department of Electrical and Computer Engineering, College of Engineering, Northeastern University, Boston, MA, United States

**Keywords:** stroke, upper limb, subacute, virtual reality, robotics, transcranial magnetic stimulation, EEG

## Abstract

**Introduction:** Innovative motor therapies have attempted to reduce upper extremity impairment after stroke but have not made substantial improvement as over 50% of people post-stroke continue to have sensorimotor deficits affecting their self-care and participation in daily activities. Intervention studies have focused on the role of increased dosing, however recent studies have indicated that timing of rehabilitation interventions may be as important as dosing and importantly, that dosing and timing interact in mediating effectiveness. This study is designed to empirically test dosing and timing.

**Methods and Analysis:** In this single-blinded, interventional study, subjects will be stratified on two dimensions, impairment level (Fugl-Meyer Upper Extremity Assessment (FM) and presence or absence of Motor Evoked Potentials (MEPs) as follows; (1) Severe, FM score 10–19, MEP+, (2) Severe, FM score 10–19, MEP–, (3) Moderate, FM score 20–49, MEP+, (4) Moderate, FM score 20–49, MEP–. Subjects not eligible for TMS will be assigned to either group 2 (if severe) or group 3 (if moderate). Stratified block randomization will then be used to achieve a balanced assignment. Early Robotic/VR Therapy (EVR) experimental group will receive in-patient usual care therapy plus an extra 10 h of intensive upper extremity therapy focusing on the hand using robotically facilitated rehabilitation interventions presented in virtual environments and initiated 5–30 days post-stroke. Delayed Robotic/VR Therapy (DVR) experimental group will receive the same intervention but initiated 30–60 days post-stroke. Dose-matched usual care group (DMUC) will receive an extra 10 h of usual care initiated 5–30 days post-stroke. Usual Care Group (UC) will receive the usual amount of physical/occupational therapy.

**Outcomes:** There are clinical, neurophysiological, and kinematic/kinetic measures, plus measures of daily arm use and quality of life. Primary outcome is the Action Research Arm Test (ARAT) measured at 4 months post-stroke.

**Discussion:** Outcome measures will be assessed to determine whether there is an early time period in which rehabilitation will be most effective, and whether there is a difference in the recapture of premorbid patterns of movement vs. the development of an efficient, but compensatory movement strategy.

**Ethical Considerations:** The IRBs of New Jersey Institute of Technology, Rutgers University, Northeastern University, and Kessler Foundation reviewed and approved all study protocols. Study was registered in https://ClinicalTrials.gov (NCT03569059) prior to recruitment. Dissemination will include submission to peer-reviewed journals and professional presentations.

## Introduction

Stroke is the leading cause of long-term adult disability (1). Independent upper extremity function is critically important to restore full independence and reduce the need for costly supportive care. Although innovative upper limb motor therapies, (2–5) have attempted to reduce upper extremity impairment after stroke, we have not made substantial improvement as over 50% of people post-stroke continue to have sensorimotor deficits that affect their self-care and ability to participate in daily activities (6). This lack of progress might be explained in part by the complexity of coordination of the multiple degrees of freedom required for normal upper limb function. Clearly, there is a need to develop more effective rehabilitation programs for the arm *and the hand* of persons with stroke.

The focus of recent rehabilitation studies has been on increasing the dosing and intensity of the interventions. Findings in a group of randomized controlled trials (7–10) indicated that the critical ingredients needed for arm and hand movement recovery may be the amount of treatment provided and the value of progressively increasing the level of difficulty of a task. The importance of dosage was further supported in a meta-analysis (11) of physical therapy interventions utilizing high repetition activities. These findings led to the growing consensus on the importance of intensity (number of repetitions per time on task) or dosage (time on task) to achieve better outcomes post-stroke. However, a recent phase 3 study of over 350 patients showed no differences among an additional 30 h of upper extremity structured task-oriented therapy, an equivalent dose of customary occupational therapy, or even a lower dose of customary therapy when these interventions were initiated 45 days post-stroke (12). Therefore, a consensus on optimal dosing in the early period post-stroke remains elusive (13).

It is apparent that it is not just dosing that needs to be considered. Several authors suggest that the timing of rehabilitation interventions may be as important as the dosing and have proposed that the dosing and timing of an intervention are not independent factors (14–16). Although the optimal time period is not clear, it has been shown that the first month post-stroke is a crucial time for synaptic plasticity where the brain is most responsive to sensorimotor input and training (14). The post-stroke sensitivity to intervention decreases as time post-stroke increases (17, 18). Both animal and human studies of early intervention reported better functional recovery [1–2 weeks in animals; 4 weeks in humans; (14, 19–21)] when training was initiated during the first month of recovery during this proposed period of intensive plasticity (14, 21–23). Thus, increasing rehabilitation treatment dose during this initial recovery period might have particularly beneficial effects. Nevertheless, most of the research on novel therapies involved subjects in the chronic phase after stroke, with a rather limited number of intervention studies available during the early and late acute phases (24).

However, contrary to these positive findings, some animal studies have shown that lesion size increased after early excessive limb training (25–27). Some human studies have also shown similar negative findings. Although, a meta-analysis of training studies showed a favorable effect on ADL resulted when augmented therapy was begun very early post-stroke (28), two studies did not find benefits from early intervention. The AVERT study was an early mobilization (<24 h) walking study and may not be an equivalent comparison (29). The Vectors study showed no differences between 2 h of early Constraint Induced Movement Therapy (CIMT) training and the control group but found less improvement with 3 h/day of early CIMT (30).

Therefore, it is particularly important to understand the impact of early motor training after stroke. Although, it has been proposed that early rehabilitation be integrated into comprehensive stroke centers to enhance care quality (31), the current state of health care delivery systems, in many countries, provides for limited in-patient rehabilitation post stroke. During this restricted time period, the focus is on ambulation and compensatory upper extremity activities necessary for activities of daily living. There is inadequate attention paid to rehabilitation and restoration of upper extremity movements so necessary for future independence in self-care activities. The conflicting evidence regarding timing, dosage and even the method of delivery of the increased dosing, robotics, virtual reality (32), or traditionally presented repetitive practice, suggests the need to examine both high volume training and method of training during the first-months post-stroke. Clearly, we do not yet have a good understanding of these relationships and their potential impact on recovery of motor function and brain reorganization.

### Study Aims and Hypotheses

This study is designed to empirically test the controversy regarding both the value of intensive, high dosage training as well as the optimal timing for intensive VR/Robotic training in the first 2 months after stroke. We are continuing an ongoing investigation into the effects of intensive task and impairment-based training of the hemiparetic upper limb with a focus on the hand using robotically facilitated rehabilitation interventions presented in virtual environments. The primary aim of this study is to test, at 4 months post-stroke, whether adding intensive VR/Robotic motor training to a patient's standard of care rehabilitation at 5–30 days post-stroke, improves functional outcomes of the hemiparetic hand when compared to initiating the additional VR/Robotic motor training 30–60 days post-stroke; thus testing the concept of optimal timing. Other comparisons include comparing Early VR to dose-matched usual care to test the potential benefits of the high-intensity VR/Robotic intervention. Comparing dose-matched usual care (10 additional hours of standard therapy) to usual care (no additional therapy) will inform about the benefits of increased dosing (Table 1). See controversies elucidated in recent literature (12, 30). We hypothesize that the outcomes will favor the early, high dosage VR/Robotic hand training group when compared to the delayed VR/Robotic training group and dose matched usual care group. The secondary aims will systematically investigate the mechanisms underlying the changes resulting from early timing of training and increased dosing, specifically changes in kinematics, kinetics, patterns of cortical somatotopic reorganization, and brain connectivity. We further hypothesize that the early, high dosage VR/Robotic hand training group will show a larger relative contribution of restored kinematic motor patterns to the overall improvement in motor function and these functional gains will be associated with the expansion of ipsilesional hand cortical somatotopy.

**Table 1:** Comparison groups.

**Group comparisons**	**Intervention period^**+**^**	**Test**
EVR vs. DVR^*^	5–30 vs. 30–60 days	Timing of a VR/robotic intervention
EVR vs. DMUC^**^	5–30 days	VR/Robotic vs. conventional intervention
DMUC vs. UC^***^	5–30 days	Dosing of an intervention

* Early Virtual Reality vs. Delayed Virtual Reality,

** Dose Matched Usual Care,

*** *Usual Care; ^+^Time Post-Stroke*.

## Methods and Analyses

### Study Design and Setting

This is a single blinded, interventional study, with four randomized, parallel arms. Patients will be recruited at Kessler Rehabilitation centers in New Jersey. This study was approved by the Internal Review Boards of Kessler Foundation, Rutgers University, Northeastern University, and New Jersey Institute of Technology.

### Randomization and Recruitment

**Figure 1:**
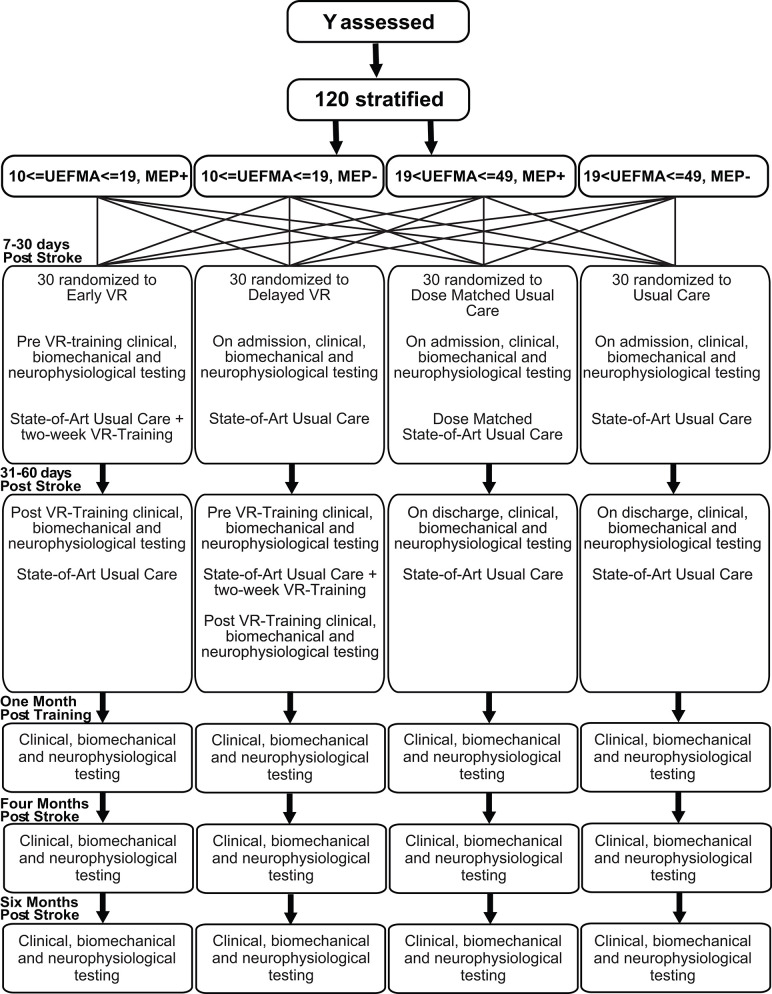
Randomization flow chart.

An independent statistician will generate the randomization sequence prior to initiation of the study. Physicians and therapists will identify potential subjects. The study coordinator will contact the patients as soon as they are medically stable, inform them about the study, screen them for inclusion and exclusion criteria and enroll them. The subjects will be stratified on two dimensions, impairment level and presence or absence of Motor Evoked Potentials (MEPs). Prior to randomization, potential subjects will be assessed using the Upper Extremity Fugl-Meyer Assessment (FM) (33) to establish motor impairment level, and Transcranial Magnetic Stimulation (TMS) for the presence or absence of MEPs in the lesioned hemisphere. These scores will be used to stratify subjects into four subgroups; (1) Severe, FM score 10–19, MEP+, (2) Severe, FM score 10–19, MEP–, (3) Moderate, FM score 20–49, MEP+, (4) Moderate, FM score 20–49, MEP-. If a subject is not eligible for TMS, he/she will be assigned to either group 2 (if severe) or group 3 (if moderate). This is based on the Stinear et al. (34) study in which study subjects with moderate impairment all had MEPs (34). Stratified block randomization will then be used to achieve a balanced assignment (35). For each of the four subgroups, every 4 subjects are one block. In each block, there is one subject for each type of treatment. One hundred twenty subjects will be assigned sequentially to four types of treatment groups (30 per group): (a) state-of-art usual care only (UC), (b) usual care plus an additional 10 h of usual care (dose-matched usual care, DMUC; initiated 5–30 days post-stroke), (c) usual care plus an additional 10 h of intensive therapy focusing on the hand using robotically facilitated rehabilitation interventions presented in virtual environments initiated **early** (5–30 days post-stroke) (EVR), and (d) usual care plus an additional 10 h of intensive therapy focusing on the hand using robotically facilitated rehabilitation interventions presented in virtual environments initiated later (30–60 days post stroke) (DVR) (see Figure 1).

### Eligibility

Studies in the chronic and early subacute phase post-stroke have consistently demonstrated the adaptability and flexibility of the *NJIT-RAVR* robotic/VR system, thus allowing for broad inclusion criteria (36, 37). We are including subjects 30–90 years old, able to follow instructions, with severe to moderate arm weakness (≥10/66 and ≤49/66 FM score), and intact cutaneous sensation. Individuals with UEFMA scores <10 will not be included as they will not have the motor ability to utilize the system effectively. Patients will be excluded from the study if they were not independent prior to the stroke, are too ill to tolerate training, have persistent motor impairment from prior stroke, have aphasia or spatial neglect precluding their performing the tasks or following task instructions, have ≥1 on the NIHSS limb ataxia item, severe proprioceptive loss, ≥3 on the Modified Ashworth Scale (of the elbow, wrist, or finger flexors), or a previous medical history of neurological deficits or orthopedic conditions that limit arm and hand movement.

### Interventions

The *NJIT-RAVR* system (38), developed by our group, is a haptic robot integrated with a library of virtually simulated rehabilitation interventions. The system will be used for the early (initiated 5–30 days post-stroke) and late (initiated 30–60 days post-stroke) intensive VR/Robotic therapy groups. This system consists of a data glove, and the Haptic Master, which is a 3 degrees of freedom admittance-controlled robot combined with a 3 degrees of freedom gimbal. The robotic arm tracks the hand in a 3D workspace and enables haptic effects, that include, springs, dampers, and other assistive and resistive forces as well as haptic objects, such as walls, floors, tables, and complex-shaped objects for hand manipulation. Th*e NJIT-Track Glove* system is an instrumented glove that tracks finger angles, a robotic hand exoskeleton that provides assistance/resistance to fingers and a three-dimensional tracking system to track hand position and orientation. Using programmable software and custom bracing of the end effector we enable use of this system for patients with a broad set of impairments and functional abilities. A library of impairment and task-based simulations have been developed. The simulations are designed to train proximal arm transport and wrist and finger manipulation. The simulations train the upper extremities either together or separately (36, 39) and include activities such as using individual fingers to play a virtual piano, extending fingers to hit a ball, transporting the arm to eliminate spaceships, using a pinch grasp to move a monkey onto higher and higher branches, integrating reach and grasp to lift virtual cups of various weights onto a haptic shelf placed at a variety of heights, and integrating reach and forearm pronation and supination to hammer a nail into a piece of wood. The subjects will participate in this training 1/h per weekday (total of 10 h).

There are three experimental groups and one comparator group. The Early Robotic/VR Therapy (EVR) experimental group will receive state-of-art inpatient usual care therapy plus an extra 10 h of intensive therapy focusing on the hand using robotically facilitated rehabilitation interventions presented in virtual environments and initiated 5–30 days post-stroke. The Delayed Robotic/VR Therapy (DVR) experimental group will receive state-of-art usual care therapy (inpatient and outpatient) plus an extra 10 h of intensive therapy focusing on the hand using robotically facilitated rehabilitation interventions presented in virtual environments and initiated 30–60 days post-stroke. The dose-matched usual care group will receive an extra 10 h of usual care physical/occupational therapy focusing on increasing range of motion, strength, and functional use of the hand. Therapists providing the dose-matched interventions will follow the same protocol the subjects receive during their usual care, and this is initiated 5–30 days post-stroke. A comparator group will receive state-of-art usual care consisting of a combination of physical, occupational, and speech therapy for 3 h each day as prescribed by Kessler Institute for Rehabilitation (KIR) physicians and regulated by the Centers for Medicare and Medicaid Services. UC consists of adaptive and progressive task and impairment based therapy including strengthening, ROM for the affected upper and lower extremity, mobility (bed mobility, transfers, gait, and elevations), and activities of daily living (dressing, toileting, meal preparation). Subjects with finger and wrist weakness typically also receive electrical stimulation of the finger and wrist extensor muscles. The same program of interventions is provided to members of the other three groups.

### Measures

There are several types of outcome measures in this study: clinical measures, neurophysiological measures, kinematic/kinetic measures, a measure of daily use of the hemiplegic extremity, and a quality of life measure. For the clinical measures, a therapist blinded to group assignment will perform all outcome assessments. For the EVR and DMUC groups, the measures will be taken immediately prior to intervention (ideally within 72 h), immediately post-intervention (ideally within 72 h), 1 month post-intervention, and 4 and 6 months post-stroke. For the DVR group, there will be an additional measurement taken immediately post-enrollment (ideally within 72 h). For the UC group, the measures will be taken immediately post-enrollment (ideally within 72 h), 2 weeks post the first test, 1 month post the second test and 4 and 6 months post-stroke.

### Primary Outcome

The primary clinical outcome for this study is the Action Research Arm Test (ARAT) (40). Four subscales in this 19-item test measure the following upper extremity activities; grasp (6 items), grip (4 items), pinch (6 items), and movement 3 items). Each item is rated on a four-point scale with the following values, 0 = no movement, 1 = the movement task is partially performed, 2 = the movement task is completed but takes abnormally long, and 3 = normal movement. Scores range from 0 to 57 with higher scores indicating better performance.

#### Secondary Outcomes – Clinical

The secondary clinical outcomes include the Box and Blocks test, Upper Extremity Fugl-Meyer Assessment, the Patients' Structured Subjective Assessment, the EuroQol Five Dimensions Test and the National Institutes of Health Stroke Scale (NIHSS).

*Blocks and Box Test (BBT)* (41) is a unilateral assessment of gross manual dexterity. It can be used for a variety of neurological diagnoses including stroke. The subject is asked to move blocks (1" cube) from one compartment of the box to another compartment of equal size. The score is the maximum number of blocks that can be moved within 60 s.

*Upper Extremity Fugl-Meyer Assessment (FM)* (33) is an impairment based upper extremity measure consisting of 33 movements that test single and multi-joint movement in and out of synergy, digit individuation, speed, dysmetria, ataxia, and reflexes. Each item is rated on a three-point scale, 0 = cannot perform, 1 = performs partially, 2 = performs fully, for a total score of 66. Higher scores indicate less impairment and more isolated motions.

*Patient's Structured Subjective Assessment* (42) will be performed for the EVR and DVR groups. This is a 27-item questionnaire that addresses the subject's perception of the function of their hemiplegic arm and the effect this intervention had on their hand function. Subjects fill out the questionnaire prior to and directly after the intervention (ideally within 72 h). Some questions require a response such as disagree, neutral and agree, others require ordering their gaming activity preferences, or responding to a question with a short answer.

*EuroQo-EQ-5Dl* (43) is a self-rated health-related quality of life measure. Respondents rate themselves on five measures; (1). mobility (or ambulation), (2). self-care (bathing and dressing), (3). usual activities (their current function in “work, study, housework, family or leisure activities,” (4). pain/discomfort, and (5). anxiety/depression. The severity of each measure is rated as no problems, slight problems, moderate problems, severe problems, and extreme problems, with a 1-digit number describing the level selected for each measure. The five measurements are combined into a 5-digit number to describe the health state of the patient.

*National Institutes of Health Stroke Scale (NIHSS*). This 15-item scale evaluates the neurological status in stroke patients. Levels of consciousness, language, neglect, visual-field loss, extraocular movement, motor strength, ataxia, dysarthria, and sensory loss are each scored. The scores for each section range from 0 (normal) to 4.

#### Secondary Outcomes-Kinematic/Kinetic

*Secondary Outcomes-Kinematic/Kinetic* include maximum finger opening, active range of motion of the upper extremity, accuracy and coordination of active movement, measurements of force and force regulation, and a measure of coordination between hand transport and grasp during a reaching movement.

*Maximum pinch, lateral pinch, and whole hand grasping forces* will be measured using an ATI Nano17 force sensor. Further, to measure the ability to regulate pinch force, square, and sine waves will be presented on a computer screen. During square wave tracking, subjects will be asked to hold force for 12 s for a single repetition, at 10, 30, 40, 20, 30% of their maximum pinch force, continuously with 4 s rest between repetitions. During sine wave tracking, subjects will be asked to constantly adjust pinch force to follow a sine wave with frequency at 0.5 Hz and amplitude at 50% of maximum pinch force. Vertical position of the cursor on the screen will be defined by isometric force between the thumb and index fingertips measured by the force sensor. Root mean square error will be calculated to assess the accuracy of pinch force control.

*Active Range of Motion for fingers, wrist, elbow, and shoulder* will be measured using the CyberGlove and an array of motion sensors (TrackStar). *Maximum Finger Opening* will be estimated as the mean of the maximum active extension of the metacarpophalangeal (MCP) and proximal interphalangeal (PIP) joints for all five fingers. *Ability to Regulate Hand Opening/Closing* will be evaluated by measuring the accuracy of tracking a sine wave on the computer screen while the vertical position of the cursor is defined by the average of four MCP joints. The ability to regulate wrist flexion/extension, elbow flexion/extension, and shoulder abduction/adduction will also be evaluated separately using similar sine wave tracking. The wrist, elbow or shoulder angle will be used to define the vertical position of the cursor on the screen.

*Real-world Reach-Grasp Test* (44) will be used to measure the kinematics of everyday movements involving grasping and manipulating household objects. The goal of this outcome measure is to analyze whether the intervention training transfers to real-world reaching and grasping patterns of inter-finger coordination during pre-shaping of the hand, coordination between hand pre-shaping and transport of the hand to the target, and coordination of upper limb segments. Kinematics of reaching for an object, lifting it from the support, transporting it to a predefined location, and releasing the object will be evaluated.

The Reach-Grasp Test will be administered to the hemiparetic arm at each test point and the non-hemiparetic upper extremity once using an optical motion capture system (Optitrack Prime 13 cameras, Optitrack, USA). *Fifteen* active makers will be placed on each fingertip, metacarpophalangeal, and proximal interphalangeal joints. Additionally, 4 passive markers will be placed on the back of the hand, elbow, shoulder, and sternum. Subjects will be seated at a table with their hips and knees in a 90 degrees position. Their semi-pronated forearm and palm will rest on the table, with the hand positioned midline between the shoulders and 10 cm away from the chest. Test objects will be positioned at subjects' midline 20 cm from the subjects' hand. Subjects will be instructed to reach for an object, lift it from the support, transport it to a predefined location and release the object. Each reach will be repeated up to 10 times based on subject tolerance. OptiTrack motion capture software (Motive) will be used to record the marker coordinates. Hand peak velocity, time to peak velocity and movement smoothness of hand spatial trajectory will be calculated separately for all three sub movements: reaching to object, transporting object, and returning arm back to the starting position. Hand preshaping during reach will be analyzed as a measure of coordination between hand transport and grasp and will be used as a secondary kinematic outcome (44). Five objects include small (1 inch) and large (3 inch) cubes, small (2.5 inch diameter), and large (4.5 inch diameter) circular objects, and a small spray bottle (4 inch height).

*Robotic kinetic measures* will be collected to compare the immediate effects of training in the EVR and DVR groups. Subjects will perform a robot-based Arm Reaching test every other day immediately prior to VR/robotic training during the 10 days of training. Subjects will hold a robotic arm to reach 5 haptically rendered spheres located in a 3D virtual environment at various heights and depths to measure changes in elbow/shoulder range, velocity, and coordination.

*Measurement of Daily Use of Upper Extremity*. Activity monitors (GT9X Link, Actigraph) will be worn by the subject on both wrists for 24 h to quantify the daily use of the affected arm.

#### Secondary Outcomes-Neurophysiological

The neurophysiological outcomes include cortical area representation of the finger-hand muscles as measured by Transcranial Magnetic Stimulation (TMS), and brain connectivity as measured through Electroencephalography (EEG). TMS mapping and EEG data will be acquired at pre- and post-therapy and at 4 months post stroke (see Table 2).

**Table 2:** Assessment schedule for the outcome measures.

**Measure**	**Domain measured**	Pre-test^**^	**Post-test**	**1 month**	**4 months**	**6 months**
**Primary outcome**						
Action research arm test	Upper limb function	x	x	x	x	x
**Secondary outcomes - clinical**						
Box and blocks	Gross manual dexterity	x	x	x	x	x
Upper extremity fugl-meyer assessment	Upper limb impairment	x	x	x	x	x
Patient's structured assessment	Perception of limb function		x			
EuroQol	Health-related quality of life	x	x	x	x	x
NIH health stroke scale	Neurological status	x				
**Secondary outcomes – kinematic/kinetic**						
Maximum isometric pinch force	Maximum force produced	x	x	x	x	x
Pinch force regulation	Modulation of force production	x	x	x	x	x
Range of motion (ROM)	Active/Passive ROM upper limb	x	x	x	x	x
Real-world reach-to-grasp test	Kinematics of grasping	x	x	x	x	x
Robot based daily kinematic measures	Immediate effects of training	x	x			
Home-based accelerometry	Amount of daily arm use		x^*^		x	
**Secondary outcomes – neurophysiological**						
TMS - MEP amplitude and extent	Patterns of cortical reorganization	x	x		x	
EEG	Resting state/task-based brain connectivity	x	x	x	x	x

* *ideally within 72 h after Post-test*.

** *The delayed VR group has two PRE tests, one in the hospital and one immediately prior to the delayed VR intervention*.

*Patterns of Corticospinal Reorganization* will be assayed using single-pulse TMS (Magstim Rapid with an AirFilm Coil). Changes in the ipsilesional and contralesional hand cortical territory will be quantified using motor evoked potentials (MEPs) in persons that meet specific safety criteria for TMS. The topographic representation of the following hand and arm muscles will be mapped before and after training and at the three retention time points: first dorsal interosseous [FDI], extensor indicis longus [EI], abductor pollicis brevis [APB], abductor minimi [ADM], flexor digitorum superficialis [FDS], extensor digitorum communis [EDC], flexor carpi radialis (FCR), extensor carpi radialis [ECR], extensor pollicis longus [EPL], and brachioradialis [BR]. To ensure spatial precision we will employ frameless neuronavigation (Brainsight, Rogue Research, Inc.).

A threshold of 50 uV will be used to detect MEPs against background EMG (45). MEP amplitude will be measured as the peak-to-peak amplitude of the EMG signal 20–50 ms after the TMS pulse. In addition, the amplitude of the background EMG will be calculated as the area under the EMG envelope in the 50 ms interval before the TMS pulse where the envelope is generated by filtering the signal with 2nd order Butterworth filter (5–250 Hz band-pass), full-wave rectifying the filtered signal and applying 20 Hz low-pass filter. MEP and background EMG amplitude will be estimated for each muscle and for each stimulation point. MEP amplitudes and coordinates of the stimulation points will be interpolated to a 7 × 7 cm mesh of 5 mm resolution centered on the M1 hotspot using cubic surface interpolation method (46, 47). This will allow comparisons across maps and sessions. The weighted-average location of corticospinal output devoted to a muscle, or center of gravity (COG), is expressed as an MEP-weighted centroid of the cortical representation in the rostral-caudal (COGx) and medial-lateral (COGy) plane using standard equations (45, 48).

The product of the number of interpolated scalp sites eliciting MEPs and the map resolution (0.5 mm) represents the extent of the representation producing corticospinal output (MEPs), or map area (47–51). Overall excitation is represented by map volume (also described in the literature as “weight”), (48, 51) and calculated as the integrated amplitude using double trapezoidal integration of interpolated maps (52) across all responsive stimulation sites (51). Map area, volume, and COG have been used extensively to describe sensorimotor cortex reorganization after stroke [for a review see Cortes et al. (53)]. The shift of the COG, enlargement of motor representation area, and the increase in the volume of excitation of the ipsilesional hemisphere have been shown to correlate with functional measures of motor recovery. The total area representing the intrinsic muscles (FDI, EI, ADM, APB) and extrinsic muscles (FDS, EDC, FCR, ECR, EPL, and BR) will be calculated to quantify changes in cortical somatotopy of fine and gross motor muscle groups of the hand, respectively.

##### Brain Connectivity

In an attempt to predict who will respond favorably to our therapeutic intervention, both Resting-State Brain Connectivity and Task-Based Brain Connectivity will be evaluated using EEG. Channels showing significant EEG desynchronization during the tasks will be identified and used in subsequent EEG connectivity analysis in Source Information Flow (SIFT) toolbox (54) to explore within-brain connectivity.

##### Experimental Conditions

The EEG-only experiments will be conducted prior to and immediately after the training, and at each of the three retention tests (see Table 2). Data will be collected (1) at rest, (2) during simple target-directed finger flexion/extension tasks of affected hand, and (3) during simple finger flexion/extension of unaffected hand with a randomized veridical or mirrored feedback of VR hand. In the resting task, 10 min of resting data will be collected while subjects are focusing their gaze on an object or cursor in the center of their visual field and relax for 10 min. In the affected hand movement task, subjects will wear data gloves that record finger joint angles. The joint angle data will be used in real-time to animate hand models presented on the computer screen. Data will be recorded as subjects perform 20 trials (5 s duration) of whole hand target-directed finger flexion and 20 trials of target-directed finger extension separated by 4–6 s of rest. The setup and trial durations are the same in the unaffected movement task, but visual feedback will randomly show two conditions, (1) veridical feedback, or (2) mirrored feedback. Due to the time limitations of inpatient participants, EEG-only experiments will be conducted using water-based caps (R-Net, Brain Products).

##### EEG Data Analysis

The EEG signals will be band-pass filtered (3–50 Hz) and decomposed into independent components using ICA. The resting state EEG data will be divided into non-overlapping 1-s epochs. The task-based EEG data will be divided into epochs that match the duration of task events. Data will be re-referenced offline to the mean signal of all 64 channels. Visual inspection will be done to remove EEG epochs and ICA components contaminated by muscle activity, or motion artifacts. The remaining ICA components will be transferred into channel space. Channel-based EEG connectivity will be studied using EEGLAB SIFT toolbox (54).

If stroke participants' MRI data is available, the stroke lesions will be mapped and a Boundary Element Model (BEM) will be created based on the subject's MRI image using FreeSurfer software, after masking out the mapped stroke lesion. Source Space data will be computed based on channel data and BEM using low-resolution brain electromagnetic tomography (LORETA) source localization method (55). Data will be extracted from selected regions of interests (ROIs) in the sensory, motor, premotor, visuomotor, and primary visual areas. Event-Related Spectral Perturbations (ERSPs) will be calculated for the selected ROIs. Between-ROI connectivity (BRC) in the brain will be quantified using normalized direct transfer function SIFT toolbox (54).

### Data Collection and Management

The assessment schedule is summarized in Table 2. Pre-post training assessments will take place at the KIR. The 1-month post therapy, 4 and 6-month post-stroke assessments will take place either at KIR, NJIT, or at Rutgers University. The clinical tests for all subjects will be assessed by an evaluator blinded to group assignment (see Table 2). Study data will be maintained in locked files and password protected computers. The code containing subject ID's will be maintained by the study coordinator.

### Sample Size

For Aim 1, the planned primary analysis method is a mixed effect model. The primary outcome measure is the change in ARAT score from baseline evaluated at 4 months post-stroke. The estimated effect size for the interaction term is 0.42 (Cohen's f), and the estimated within-subject correlation is 0.7. For testing the hypothesis of Treatment Group (EVR, DVR, UC, DMUC) ^*^Test Time (Baseline, 4 M post-stroke, 6 M post-stroke) effect, and assuming compound symmetry correlation for within-subject measurements, for significance level 0.05, a total of 84 patients (21 for each group) are needed to detect the overall Treatment Group ^*^ Test Time interaction effect. But for the main planned contrasts, Treatment Group (EVR, DVR) ^*^ Test Time (Baseline, 4 M post-stroke) and Treatment Group (UC, DMUC) ^*^ Test Time (Baseline, 4 M post-stroke), a total of 96 patients (24 for each group) are required to detect the effect size of 0.42 between EVR and DVR or between UC and DMUC over time. Since detecting these contrasts is the main goal of the study, at least 96 subjects, 24 subjects per group, are required. After taking into account subject attrition, our total sample size will be equal to 120. We plan to run an interim analysis and update the sample size when we have recruited 50% of subjects.

### Statistical Analysis

The primary aim of the study is to test, at 4 months post-stroke, whether intensive VR/Robotic motor training initiated 5–30 days post-stroke, improves functional outcomes of the hemiparetic arm and hand when compared to VR/Robotic motor training initiated on 30–60 days post-stroke. Other comparsions include evaluating the type of training (EVR vs. DMUC) and dosing (DMUC vs. UC) initiated within 5–30 days post-stroke. The secondary aims will systematically investigate the mechanisms underlying the changes resulting from the early timing of training and increased dosing. Aim 2 will quantify the recovery and compensation profiles by examining the changes in kinematics and kinetics of the hand and arm associated with this training and Aim 3 will quantify patterns of cortical somatotopic reorganization and will investigate the relationship among these patterns of reorganization and the clinical and biomechanical outcomes.

We will use the robust mixed effect model approach to analyze the data. We demonstrated in the past that this analytic approach is robust to detect treatment effects, even with relatively small groups of patients in whom within-group variance occurs with respect to stroke characteristics (56). Test Time (Pre, Post, 1-month post-treatment, 4 months post-stroke, 6 months post-stroke) and Treatment Group (EVR, DVR, DMUC, UC) will be treated as fixed effects in the mixed models. The interaction term between Test Time and Treatment Group will be included. Baseline measurements will be added as a covariate into mixed models for outcome variables. Random intercept will be used to model subject-level variation. Covariance pattern and model selection will be implemented with likelihood-based tests. Contrasts to compare group means will be applied to obtain comparisons between time, and between treatments, with multiplicity adjustment considered. We have an a priori interest in specific Test Time by Treatment Group interaction effects for the EVR-DVR, for the DMUC-UC, and for the EVR-DMUC comparisons. The particular differences we are interested in are Pre vs. Post and Pre vs. second (4 months post stroke) retention assessments. In the case of balanced data and equal variances, we will use the Tukey procedure to test for significant interaction effects, otherwise, Bonferroni correction will be used. In our design, the timing comparison is the difference between EVR and DVR. The dosage comparison is the difference between UC (usual care) and the DMUC (usual care plus 10 additional hours). In addition, we will be able to compare the type of training effect, VR vs. dose-matched usual care (EVR vs. DMUC), where both types of training are delivered in the same time period.

#### Primary and Secondary Outcomes – Clinical

Our overall primary outcome measure will be the ARAT score. Additional secondary outcome measures for Aim 1 will be Box and Blocks and the Upper Extremity Fugl-Meyer Assessment, the Patients' Structured Subjective Assessment questionnaire, the EuroQol Five Dimensions Test and the National Institutes of Health Stroke Scale (NIHSS).

#### Secondary Outcomes - Kinematic/Kinetic

For Aim 2 our initial statistical analysis will be applied to the vector of z-normed kinematic measures [Pinch Force Tracking Error, Maximum Pinch Force, Finger Flexion/Extension Tracking Error, Maximum Finger Opening], with fixed effects of Test Time and Treatment Group (EVR, DVR, DMUC and UC). *Post-hoc* comparisons will be analyzed through pre-planned contrasts. Separate analyses will be conducted for each output variable if appropriate. As exploratory analyses, the relationship between multiple output variables will be investigated using multiple-output-stacked mixed effects models.

Grasping of objects of various sizes and shapes will be analyzed (44). The subjects' ability to preshape their hands as it approaches the object to be grasped will be evaluated using support vector machine (SVM)-based classification analyses of the finger joints. Flexion/extension in the metacarpophalangeal and peripheral interphalangeal joints of the fingers plus finger abduction angles will be used as input variables. At each moment during reaching, the classification algorithm will predict which of the objects the subject is grasping in this particular trial. Classification error will be calculated as percent of trials where the algorithm made an incorrect prediction and will serve as an outcome measure, with smaller error values indicating better hand function. In addition to SVM, other classification methods will be investigated (e.g., linear discriminant analysis). The time profiles of the classification errors for grasping with the affected arm will be analyzed longitudinally. The time profiles of the magnitude of this classification error will be analyzed with mixed model analysis with fixed effects of Treatment Group (EVR, DVR, DMUC, UC), Test Time, Condition (either Object Size: Small, Large; or Object Shape: Round, Rectangular, Irregular) and Movement Time (5–95% epochs). Finally, classification error profiles obtained during grasping with the affected arm will be compared with the profiles of the less affected arm.

#### Secondary Outcomes - Neurophysiological

For Aim 3, variables will be analyzed with a mixed model, with fixed effects of TEST TIME (Baseline, Post, 1, 4, 6 M), sensorimotor cortex (Ipsilesional, Contralesional), Muscle (Intrinsic, Extrinsic) and Treatment Group (EVR, DVR, DMUC, UC). AREA (size of the cortical surface area in the affected hemisphere where MEPs can be elicited, to be used as an index of focusing of the cortical excitability distribution) will be used as an outcome measure. In addition to group analyses, we will use a Spearman rank order test to correlate individual subject measures of cortical topography (Area/Volume/COG shift, measured by TMS mapping) and clinical measurements of impairment (hand/wrist UEFMA) and function (distal WMFT) across testing time points. In our experience, individual subject data analysis has proven invaluable in understanding the effects of therapy on functional recovery. Non-parametric statistics (Kruskal-Wallis or Mann-Whitney) will be used for variables that fail tests of normality. Alpha level will be set at 0.05. Tukey *post-hoc* tests will be used to investigate significant interactions.

Lesion size and location (cortical/subcortical) will be used for secondary analyses regarding responders/non–responders, etc. (56–58) in which we used theory-based categorization of brain lesions to determine regions associated with better response to stroke rehabilitation, using a mixed-model analysis.

#### Multivariate Regression Analyses

In addition, we will conduct hypothesis-generating multivariate regression analyses of the outcomes. For those models, the independent variables will consist of the kinematic/kinetic and neurophysiological scores, and the dependent variable will be the ARAT, Fugl-Meyer, or BBT score. We will also conduct separate regression models for each of the dependent variables. While modeling potential intrinsic correlation between VR measurements taken on the same person via multivariate models, potential collinearity among covariates will be investigated and appropriate regularization will be implemented accordingly. Collinearity analysis may also inform us whether some VR games might be redundant, and aid in future development of VR therapies. Finally, suitable contrasts for hypotheses related to comparing VR performance score among the first and last 2 days of therapy, and the two retention tests will be considered.

#### Management of Statistical Assumptions

For all analyses, model-related assumptions will be examined. For example, in case that normality assumption required by linear mixed effects model cannot be met, appropriate transformation to center the dependent variables will be first sought. If no appropriate transformation is applicable, non-parametric mixed models will be applied. Collinearity between covariates will be examined with variance inflation factor (multilevel variance inflation factor to the case of mixed effects models). Appropriate selection from collinear covariates will be based on knowledge or literature. Statistical methods like principal component analysis will be used to extract factors from collinear covariates when needed.

#### Management of Missing Data and Dropouts

Besides listwise exclusion, which assumes missing data completely at random, multiple imputation methods will be used as a sensitivity analysis to examine the effect of different assumptions about missing data.

### Current Results

Currently, there are seven subjects who have met the eligibility criteria, completed the training or usual care, and had their 6 month retention tests. The screening for patients is on-going. The estimated enrollment is 25–30 patients/year.

### Anticipated Results

Clinical outcome measures will be assessed in combination with the kinematic/kinetic and neurophysiological measures to determine whether; (1) early intensive training that focuses on the hand results in a more functional hemiparetic upper extremity, (2) there is an optimal early time period in which rehabilitation will be most effective, (3) it is necessary to initiate intensive arm and hand therapy during the early inpatient rehabilitation phase or will comparable outcomes be achieved if therapy is initiated right after discharge and, (4) there is a difference in the recapture of premorbid patterns of movement vs. the development of an efficient, but compensatory movement strategy.

## Discussion

This study focuses on arm and hand rehabilitation that (1) can be delivered in an inpatient rehabilitation facility during the early period of heightened neuroplasticity using VR/robotic simulations to facilitate the delivery of increased intensity of an intervention, (2) can quantify intensity to distinguish between total therapy time and actual movement time, (3) can specifically target the particular movement deficits that need to be modified, and (4) can incorporate patients with minimal movement. Independent upper extremity function is critically important to restore full independence and reduce the need for costly supportive care. One of the issues that may contribute to less than satisfactory outcomes for the hand (as well as the arm) is the complexity of hand motor control that involves coordination of multiple degrees of freedom. Early hand therapy is often limited when patients have not recovered sufficient motor function to utilize the hemiparetic hand during functional activities. Frequently therapists train compensatory movements using the less affected upper extremity, in an effort to attain maximal independence prior to patient's discharge in the face of the ever-decreasing length of stay in inpatient rehabilitation as part of a stroke care plan. This VR/robotic system is well-suited for the delivery of hand and arm training for more affected patients. Sensory and perceptual affordances provided by the integration of VR and robotics can target the unique hand deficits that one cannot address in real-world therapy (37), and thus possibly allow for functional improvement to move beyond the spontaneous recovery predicted in the literature (62).

The *NJIT/RAVR* and *NJIT-Track Gloves* systems have adaptive algorithms to drive individual finger movement, can modify the workspace to increase range of motion and can provide gain modification in order to allow a patient with a minimal amount of hand movement to interact successfully with the virtual reality simulations (37). Evaluating treatment response can be substantially improved with the addition of kinematic analyses (59). Currently used clinical assessments provide uncertain measures of a patient's progress and improvement due to a lack of sensitivity and potential ceiling effects. Multiple sources indicate that analysis of treatment response and understanding of recovery and compensatory processes can be substantially improved by incorporating analyses of kinematic/kinetic variables into clinical research studies (60). Measures that focus purely on task completion do not discriminate between neural recovery and functional compensation (16). Several authors suggest that kinematic analysis may provide a more effective way to measure the recovery of motor function following stroke (59, 61). More specifically, a lack of longitudinal analysis of 3D kinematics of functional upper extremity movements during early stroke recovery limits our true understanding of what motor changes actually occur and what patients actually learn during the early intervention period (61).

We hope that TMS and EEG will increase the ability of our study to generate translational, neurophysiologic data. We will quantify and relate neural reorganization of the corticospinal system and the cortical networks in both ipsilateral and contralateral hemispheres with behavioral (clinical and kinematic) recovery during the first 6 months post-stroke. While neurophysiological measures are considered promising biomarkers of corticospinal integrity and recovery, there are few studies utilizing these approaches to date (62, 63) and there has been no single well-controlled study examining changes in the trajectory of neural recovery subsequent to intensive, progressive therapy initiated at different stages, early post-stroke.

At the end of this project, we will characterize the effects of dosing and timing and determine whether (1) there is an optimal early time period in which rehabilitation will be most effective and when intensive training focused on the hand will result in a more functional hemiparetic arm, (2) it is necessary to initiate intensive hand therapy during the very early inpatient rehabilitation phase or will comparable outcomes be achieved if the therapy is initiated right after discharge, in the outpatient period when the cost of care is less, (3) early training results in a difference in the recapture of premorbid movement patterns vs. the development of an efficient, but compensatory movement strategy, and (4) whether patterns of cortical reorganization explain the prognosis for recovery. However, we will not be able to investigate the timing by dosing interaction and this is a limitation of the study.

The findings from Aim 1 will advance our understanding regarding the optimal time frame for the initiation of intensive rehabilitation. Knowledge gained in Aims 2 and 3 will provide a window into the mechanism of action underlying a rehabilitation intervention by advancing our understanding of the kinematic and neurophysiologic mechanisms underlying the recovery process mediated by this intensive and progression intervention. Importantly, we will have provided much-needed data for clinicians on the optimization of the timing of therapeutic interventions. This information will be relevant whether the rehabilitation is performed using more traditional means or whether it delivered through newer technology. Optimizing rehabilitation strategies and providing a means to deliver the intensity and progression of practice required for modifying neural architecture opens the window to potential new methods of rehabilitation. The idea that therapy can be used as a vehicle for driving neural reorganization is relatively new and will serve as a foundation for developing further novel therapeutic interventions based on neuroscientific principles.

It is evident that providing additional, intensive therapy during a stay in an inpatient rehabilitation facility is more complicated to implement, and difficult for patients to tolerate, than initiating it in the outpatient setting, immediately after discharge. To date, we have successfully integrated intensive hand therapy into an inpatient rehabilitation protocol. The next step in this line of inquiry will focus on developing an understanding of the interactions occurring between 3 critical elements: (1) a focus on the recovery of hand function, (2) facilitation of progressive and intensive training through the use of robotically facilitated, virtually simulated rehabilitation interventions, (3) beginning training early after stroke, during the period of heightened neuroplasticity. This study will fill a critical gap in the literature and make a significant advancement in the investigation of putative interventions for recovery of hand function in persons with stroke.

## Ethical Consideration

The IRB's of New Jersey Institute of Technology, Rutgers University, Northeastern University, and the Kessler Foundation, reviewed and approved all study protocols. They all require annual updates of the study and monitor for adverse events. We appreciate the importance of accurately monitoring data collection as well as the possibility of the occurrence of an unexpected adverse event. To deal with these issues we have included a data and safety monitoring board (DSMB) which consists of a Chair and 3 members all administratively independent of the study and the Kessler Foundation. The DSMB will assist the IRB's and the study personnel in the careful monitoring of the risk/benefit ratio of the conduction of this study The DSMB will meet regularly to ensure the safety of the participants during the course of the study and the validity and integrity of the data. They monitor patient safety recruitment, adherence to inclusion and exclusion criteria, retention, deviations from assigned treatments, quality control, and interim analyses of primary and main secondary outcomes as well as the occurrence of adverse events and other indicators of patient safety. To provide an extra level of oversight in regard to participant safety, there is a Medical Monitor who takes primary responsibility for deciding management for study adverse events. All adverse events and participant dropouts will be reported to the PIs immediately so the case can be examined in detail with the on-site Medical Monitor to determine the reason for drop out and/or circumstances behind the adverse event. Should any adverse events deemed to increase risks to subjects be identified, the study will stop immediately, and an investigation will be conducted. All serious adverse events will be reported immediately to both Rutgers University Human Subjects Protection Program, The New Jersey Institute of Technology IRB, and the Kessler Foundation IRB according to the policies of each institution. The study coordinator will monitor ongoing data collection, check actual participant files for recording accuracy, and monitor data entry for accuracy, including random checks throughout the duration of the study.

## Ethics Statement

The studies involving human participants were reviewed and approved by the IRB committees of New Jersey Institute of Technology, Rutgers University, Northeastern University, and Kessler Foundation. The patients/participants provided their written informed consent to participate in this study.

## Author Contributions

ASM, ET, GF, QQ, JP, MY, and SA were involved in designing the study protocol. Manuscript writing was performed by ASM and SA equally, and revised by JP, MY, AB, KN, ET, QQ, ASM, AJM, and GF. All authors contributed to the article and approved the submitted version.

## Conflict of Interest

The authors declare that the research was conducted in the absence of any commercial or financial relationships that could be construed as a potential conflict of interest.
